# Two Novel Mutations of the *NPM1* Gene in Syrian Adult Patients with Acute Myeloid Leukemia and Normal Karyotype

**DOI:** 10.31557/APJCP.2021.22.1.227

**Published:** 2021-01

**Authors:** Ismael F. Alarbeed, Abdulsamad Wafa, Faten Moassass, Balssel Al-Halabi, Walid Alachkar, Imad Aboukhamis

**Affiliations:** 1 *Department of Microbiology, Hematology and Immunology, Faculty of Pharmacy, Damascus University, Ministry of High Education, Damascus, Syria. *; 2 *Department of Molecular Biology and Biotechnology, Human Genetics Division, Atomic Energy Commission, Damascus, Syria. *

**Keywords:** Acute myeloid leukemia (AML), normal karyotype, NPM1 gene mutation, prognositic factor

## Abstract

**Objective:**

Somatic mutations in exon 12 of the *NPM1* gene is one of the most common genetic abnormalities in adult acute myeloid leukemia (AML), which is observed in 25-35% of AML patients and in 50-60% of patients with cytogenetically normal AML (CN-AML).

**Methods:**

We performed Sanger sequencing of exon 12 of the *NPM1* gene, on 44 CN-AML patients to characterize NPM1 status.

**Results:**

In this study, NPM1 mutations were identified in 10 (22.7%) of the 44 CN-AML patients. Among the 10 patients with NPM1 mutations, type A NPM1 mutations were identified in 8 (80%) patients, whereas non-A type NPM1 mutations were observed in 2 (20%) patients. Two non-A type NPM1 mutations were not previously reported: c.867-868InsCGGA and c.861-862InsTGCA. These two novel mutant proteins display a nuclear export signal (NES) motif (L-xxx-L-xx-V-x-L) less frequently and L-x-Lx-V-xx-V-x-L it has been never seen before, yet. However, both novel mutations show a tryptophan loss at codon 288 and 290 at the mutant C-terminus which are crucial for aberrant nuclear export of NPM into the cytoplasm.

**Conclusions:**

This study suggests previously unreported NPM1 mutations may be non-rare and thus additional sequence analysis is needed along with conventional targeted mutational analysis to detect non type-A NPM1 mutations.

## Introduction

Acute myeloid leukemia (AML) is a heterogeneous hematologic malignancy, which is characterized by uncontrolled proliferation of hematopoietic stem cells resulting in abnormal accumulation of myeloblasts. According to 2016 World Health Organization (WHO) classification AML has several cytogenetic and molecular subgroups (Arber et al., 2016). Cytogenetic and molecular findings help clinicians to stratify AML patients and plan therapeutic strategies. As far as cytogenetic abnormalities are concerned, the prognosis of AML patients was categorized into three risk groups: good, intermediate, and poor (Grimwade et al., 1998). However, approximately 50% of AML patients show up with a cytogenetically normal (CN), representing a diverse subset of patients which are usually classified into an intermediate risk group (Gregory et al., 2009). 

Nucleophosphomin (NPM1) is a nucleocytoplasmic shuttling protein that plays an active role in ribosomal protein assembly, chromatin remodeling, DNA repair, replication, and transcription (Lindstrom 2011). Mutations in the *NPM1* gene have been reported in 25-35% of adult AML cases (Verhaak et al., 2005), which is a higher frequency 50-60% in CN-AML patients (Falini et al., 2005). However, more than 50 different mutations involving exon 12 of the *NPM1* gene have been identified in AML. All of them lead to frameshift and elongation of the protein, which is aberrantly retained in cytoplasm (Falini et al., 2005). The presence of *NPM1* mutations predict good response to induction therapy and it was associated with favorable outcome, increased complete remission (CR) rates, event-free survival (EFS) and overall survival (OS) (Liu et al., 2014). 

The most common mutation in *NPM1 *patients is type A, which duplicates a TCTG tetranucleotide in the reference sequence at 956-959 and it accounts for up to 80% of adult *AML* with *NPM1* mutations (Koh et al., 2009). Several studies have suggested that non- A type *NPM1* mutations may function as prognostic factors for poor clinical outcomes (Koh et al., 2009; Park et al., 2012). Therefore, it may be important to identify and characterize *NPM1* mutations at the nucleotide and amino acid levels. 

In this study, we evaluated for the first mutational spectrum of *NPM1* mutations in adult CN-AML Syrian patients newly diagnosed, which was directly sequenced. The biological and clinical features and the prognostic significance were also assessed.

## Materials and Methods

*Subjects*

A total of 77 patients newly diagnosed with de novo AML between February 2018 and April 2019 were included in the study. Patients without previous treatment were included in the study; patients with normal karyotype were selected for molecular analysis and patients with history of exposure to chemotherapy/radiotherapy, and secondary AML patients, were excluded. AML diagnosis was made according to French-American-British (FAB) classification. Their initial bone marrow (BM) or peripheral blood (PB) samples were collected for use in the study. Patients consisted of 41 men and 36 women; the median age was 35.2±12.4 years (range, 18-77 years) (Table 1). This study was approved by the Ethics Committee in Syrian Ministry of High Education and written informed consent was obtained from all the participants.

*Treatment Protocol*

All patients received (3+7) standard induction chemotherapy, which consisted of daunorubicin at 45 mg/m2 for 3 days and cytarabine at 100-200 mg/m^2^ for 7 days, followed by high doses of a cytarabine-based consolidation phase (cytarabine at mg/m^2^ 3 every 12 h for 3 days, repeated for 2 to 3 cycles). Patients with acute promyelocytic leukemia (M3) received all-trans retinoic acid plus anthracycline. Patients received conventional induction chemotherapy and were followed for 7.4±3.7 months. BM aspiration was performed between 21 and 28 days after initiation of chemotherapy. The patients were followed up once every 3 months with clinical examination and complete blood counts. A BM aspiration was performed if there was any suggestion of relapse on clinical examination or PB smear. 

*Cytogenetic and molecular cytogenetic analyses*

Chromosome analysis using GTG-banding was performed on BM sample prior to chemotherapy according to standard protocols (Alachkar et al., 2007). A normal male karyotype was diagnosed. Fluorescence in situ hybridization (FISH) using specific probes to detect translocations t(8;21), t(15;17), t(16;16), t(12;21), and deletion del(13q), were performed with standard method to excluded patients with chromosomal abnormalities, as previously reported (Alachkar et al., 2007).

*Analysis of the NPM1 exon 12 mutations*

Genomic DNA was isolated from PB or BM samples from all 44 de novo AML patients using the QIAamp DNA Blood Mini kit (Qiagen, Germany) according to the manufactures instructions and was stored at -20°C. The exon 12 of the *NPM1* gene was amplified using specific primer NPM1-F 5’-TTAACTCTCTGGTGGTAGAATGAA-3’ and NPM1-R 5’-CAAGACTATTTGCCATTCCTAAC-3’. The PCR reaction was performed in a total volume of 50 μl containing 200 ng of genomic DNA, 10x PCR buffer (100 mM Tris-HCl, pH 8.8, 500 mM KCl), 2 mM MgCl_2_, 200 μM dNTPs, 10 pM of each primer, and 1 U of Taq DNA polymerase. PCR conditions included initial denaturation at 95 °C for 5 min followed by 40 cycles of 94°C for 30 s, 57°C for 60 s, and 72°C for 75 s with final extension at 72°C for 5 min. The PCR products were purified and directly sequenced with reverse primer NPM1-R2 5’-GGCATTTTGGACAACACA-3’ using the ABI Prism 310 genetic analyzer (Applied Biosystems, Foster City, CA, USA). The cycle-sequencing reaction was performed in a 10-μl volume containing 1 μl of the terminator ready reaction, 5 pmol of either the forward or reverse primer and 10 ng of purified PCR product (ExoSAP-IT kit; Amersham BioSciences, Piscataway, NJ, USA). The thermal cycle protocol was 95˚C for 4 min followed by 30 cycles at 96˚C for 10 sec, 50˚C for 5 sec and 60˚C for 4 min (ABI GeneAmp PCR System 9700, Applied Biosystems). Centri-Sep columns (Princeton Separations, Adelphia, NJ, USA) were used for the effective and reliable removal of excess dye terminators (DyeEx 2.0, Qiagen, Germany) from completed DNA sequencing reactions. Data were compared and aligned with different sequences using the NCBI BLAST Assembled Genomes tool (http://blast.ncbi.nlm.nih.gov/Blast.cgi).

*Statistical Analysis*

The comparison of qualitative data such as age, WBC count, platelet count, hemoglobin level and blast count percentage between NPM1wt and NPM1mut patients were statistically evaluated using Fisher exact and chi-square tests. Overall survival (OS) was estimated for patients who received at least one induction course of therapy using the Kaplan-Meier method. P values <0.05 were considered significant. All analyses were performed using SPSS Statistics 19 software (SPSS, Chicago, IL, USA).

## Results

A total of 77 newly diagnosed adult AML patients, the median age at diagnosis was 35.2±12.4 years (18–77 years) were included in this study. Forty-four patients (57.1%) showed a normal karyotype (NK) and 33 patients had chromosomal aberrations (42.9%). *NPM1* gene mutations were studied only in AML patients with NK. However, *NPM1* gene mutations were identified in 10 of the 44 CN-AML patients (22.7%) (Table 2). Regarding FAB subtype of* AML, NPM1* mutations were found more frequently in M4 and/or M5 (80%) than in the other subtypes of AML (P = 0.04). Furthermore, the incidence of NPM1mut was significantly higher in older patients than young (40.7 vs. 33.6 years, P = 0.1). *NPM1* mutations were found more frequently in male (60%) than female patients (40%), but this difference was not significant (P =0.6).

**Figure 1:**
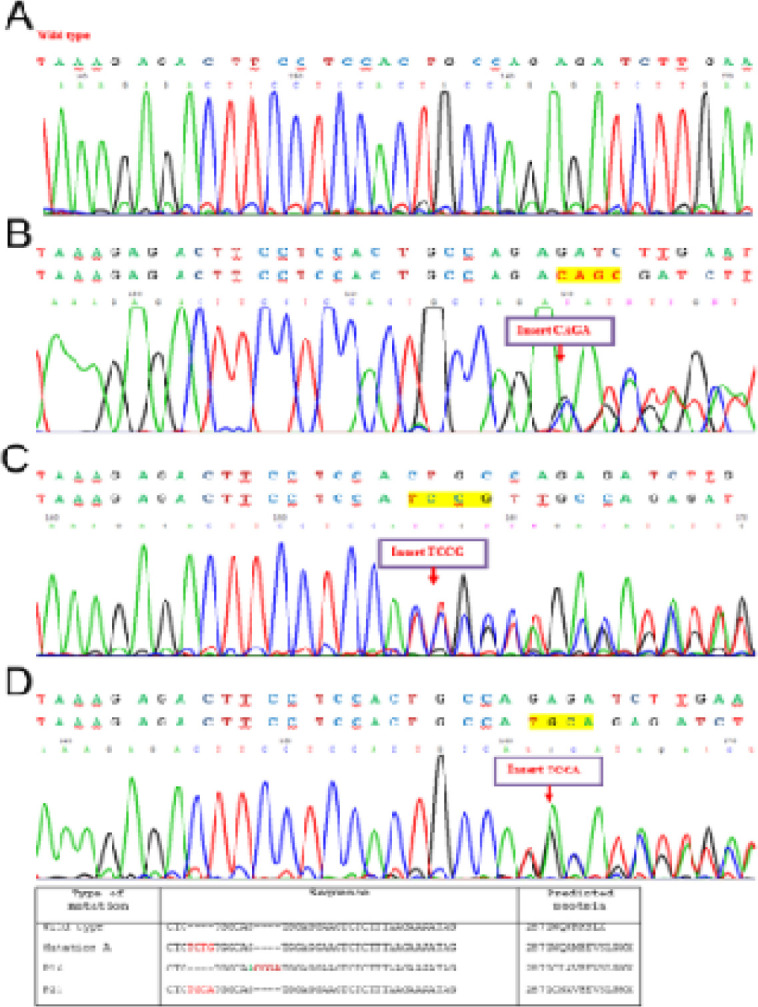
Nucleotide and Amino Acid Sequences of the Two Novel *NPM1* Mutations. (A) Wild type of *NPM1* sequence, (B) *NPM1 *mutation type A (c.860-863dupTCTG), (C) c.867-868InsCGGA (revers) (D) c.861-862InsTGCA (revers).

Of the mentioned 10 patients, type A mutation (NM-002520.5) (c.860-863dupTCTG) was identified in eight patients (80%), and *type Q* mutation consisting of a 4-bp insertion between positions nt 964 and nt 965 (NM-002520), was identified in two patients (20%): c.867-868InsCGGA (patient 16) and c.861-862InsTGCA (patient 21) (Figure 1). The predicted amino acid sequences were 287-LCLAVEEVSLRKX (patient 16) and LCNAVEEVSLRKX (patient 21), respectively, instead of wide-type sequence 287-LWQWRKSLX (Figure 1).

However, there were no significant differences between NPM1mut and NPM1wt patients with respect to age, sex, hemoglobin, platelet counts and percentage of bone marrow blasts (Table 2). However, the mean WBC count at presentation in NPM1mut was significantly higher than NPM1wt patients (76.2 vs. 31.7; p = 0.04). 

**Figure 2:**
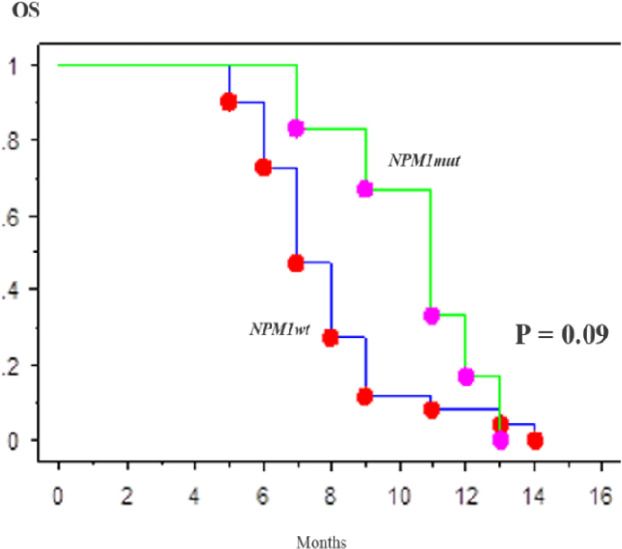
Kaplan-Meier Overall Survival Curves. OS of patients with CN-AML according to* NPM1*mut and *NPM1*wt

Of all 44 patients who received standard induction chemotherapy, 39 patients (88.6%) achieved CR. The CR rate was higher in patients with NPM1mut than NPM1wt but the difference was not significantly (p = 0.9). Moreover, of all patients who achieved CR, patients with NPM1mut had a higher relapse rate and a long survival than patients with NPM1wt (relapse rate: 30% vs. 17.7%, p = 0.08; OS rate: 7.3 vs. 6.9 months, p = 0.09). However, these differences were not statistically significant. NPM1mut were not independently associated with EFS or OS (Figure 2). In addition, a better median OS was observed in NPM1mt type-A compered NPM1mt non type-A patients when treated with intensive chemotherapy (9,9 months vs. did not reach the median; p=0.00) (data not shown).

**Table 1:** Demographic and Laboratory Data of A Do Novo Syrian Acute Myeloid Leukemia Patients

Parameters	Value
Gender	
Male	41 (53.2%)
Female	36 (46.8%)
Sex ratio (M/F)	1.2
Age (median, range)	35.2±12.4
FAB classification	
M0	1 (1.3%)
M1	10 (13%)
M2	13 (16.9%)
M3	17 (22%)
M4	25 (32.5%)
M5	10 (13%)
M6	1 (1.3%)
WBC, x 10^9^/l (median, range)	44.02 (0.8-300)
Hb, g/dl (median, range)	8.7 (3.5-16.7)
Plt, x 10^9^/l (median, range)	82.4 (16-309)
BM Blasts,%	70 (42-94)
Cytogenetic status	
Normal karyotype	44 (57.1%)
Abnormal karyotype	33 (42.9%)
Cytogenetic risk	
Favorable	11 (14.3%)
Intermediate	59 (76.6%)
Adverse	7 (9%)
*NPM1* gene mutation status	
Normal *NPM1* wide type	34 (77.3%)
Mutant *NPM1 *	10 (22.7)

FAB, French-American-British classifications; WBC, White blood cells; Hb, hemoglobin; Plt, Platelets; BM, bone marrow; CR, complete remission

**Table 2:** Clinical Patients Characteristics According to *NPM1* Status in Syrian AML Cytogeneticlly Normal Patients

Features	*NPM1* Mutant group	*NPM1* wide-type group	P value
Patients no (%)	10 (22.7%)	34 (77.3%)	
Gender			
Male	6 (60%)	19 (55.9%)	0.6
Female	4 (40%)	15 (44.1%)	
Sex ratio (M/F)	1.5	1.2	
Age (years)			
Mean	40.7±13.3	33.6±11.8	0.1
range	18-64	18-57	
WBC, x 10^9^/l			
Median	76.2±97.1	31.7±29.8	0.04
Range	7.9-300	0.8-150	
Hb (g/dl)			
Median	8.8±1.5	8.6±2.7	0.9
Range	6-10.8	3.5-16.7	
Plt x 10^9^/l			
Median	75.6±27.4	82.4±59.1	0.8
Range	37-110	17-309	
BM Blasts,%			
Median	72.2 ±12	68.5 ±15	0.2
Range	60-94	42-90	
FAB:M4&M5/others	8 (80%)	15 (44.1%)	0.04

FAB, French-American-British classifications; WBC, White blood cells; Hb, hemoglobin; Plt, Platelets; BM, bone marrow; CR, complete remission; P ˂ 0.05 is considered significant

## Discussion

We report here for the first time two novel *NPM1 *mutations and frequency, distribution pattern and clinical impact in 44 adult Syrian patients with newly diagnosed CN-AML. 

The frequency of *NPM1* mutation in Asian AML series varied between 20-32% (Suzuki et al., 2005; Chou et al., 2006; Yan et al., 2007a; Yan et al., 2007b; Boonthimat et al., 2008; Ruan et al., 2009). Whereas, it accounts in adult AML-patients of other populations between 21 and 25% (Roti et al., 2006; Lin et al., 2006; Mori et al., 2007; Ahmad et al., 2009), while reports from Thailand, China and the most European countries were between 26 and 35% (Falini et al., 2005; Yan et al., 2007a;Yan et al., 2007b;Boonthimat et al., 2008). However, in this study incidence of *NPM1* mutation in adult AML patients was 22.7%; our findings were closer to observations from India (21%) (Chauhan et al., 2013), Egypt (21.8%) (Zidan et al., 2013), and Iran (20.8%) (Rezaei et al., 2017) and in agreement with other previous studies (Roti et al., 2006; Lin et al., 2006; Mori et al., 2007; Ahmad et al., 2009). The differences in those results may be explained due to sample sizes as well as geographic and ethnic background of the studied populations.

Furthermore, the current study is in agreement with previous reports, which suggested that patients had higher WBC count in mutant versus wild type group (P = 0.04) (Chou et al., 2006; Döhner et al., 2005) but no significant differences with regard to hemoglobin level and platelets count (Lin et al., 2006 ); high incidence of mutation in *AML-M4/M5* subtype (P = 0.04) was also observed (Chou et al., 2006; Schnittger et al., 2005; Garzon et al., 2008). 

Regardless of the *NPM1* mutations type, type A mutation is the most common change, it accounts in up to 80% of adult AML patients and it has the NES motif L-xxx-V-xx-V-x-L (Koh et al., 2009). The mutations are characterized by frameshift insertions in the region encoding the C-terminus of the protein, leading to the disruption of tryptophan residues 288 and 290 and the generation of an additional NES motif, which ultimately leads to the cytoplasmic localization of the NPM1-mut as well as NPM1-wt proteins (Falini et al., 2005; Falini et al., 2006; Mariano et al., 2006). However, NPM1mut encodes cytoplasmic NPM1, which acts as an oncoprotein (Chou et al., 2006). Interestingly, we identified two novel *NPM1* mutations of type Q never been reported before yet (according to COSMIC database for somatic samples from hematopoietic and lymphoid tissue), both novel *NPM1* mutations that were identified in this study have a rare NES motif L-xxx-L-xx-V-x-L (Falini et al., 2006; Mariano et al., 2006) and L-x-Lx-V-xx-V-x-L it has been never seen before, yet. The common NES motif requires the loss of both tryptophans 288 and 290 to be transported out of the nucleus efficiently (Falini et al., 2006). However, both novel mutations in our study show a tryptophan loss at codon 288 and 290 at the mutant C-terminus which are crucial for aberrant nuclear export of NPM into the cytoplasm.

Two studies suggested that a type A mutant impacted patient prognosis favorably (Garzon et al., 2008; Hollink et al., 2009). Conflicting findings have been reported in the relationship between *NPM1 non-A* mutations and prognostic (Chou et al., 2006; Ahmad et al., 2009; Ahmad et al., 2010; Pastore et al., 2014; Hollink et al., 2009). Several studies did not demonstrate any differences in outcomes between the different types of *NPM1* mutations in pediatric AML (Ahmad et al., 2010; Pastore et al., 2014; Hollink et al., 2009). Whereas, some authors showed that patients with non-A mutations have an adverse impact on survival (Chou et al., 2006; Ahmad et al., 2009). Recent study reported that high variant allele frequency of NPM1 predict poor outcomes in de novo AML; and this effect was not affected by FLT/ITD (Patel et al., 2019). 

Regarding to the difference between NPM1mut type-A and NPM1mut non- type-A patients with achievement of CR and OS. However, no significant differences between NPM1mut type-A and NPM1mut non type-A patients was reported with regard to CR and OS (Balatzenko et al., 2014; Boissel et al., 2005). On the other hand, non-A-type NPM1mut patients were associated with a significantly shorter CR rate and shorter OS compared with NPM1wt and A-type NPM1mut (Ahmad et al., 2009). We noted similar findings as reported in (Ahmad et al., 2009), patients with NPM1mut type-A had a better OS than NPM1mut non type-A (9.9 months vs. did not reach the median; p=0.00); still, our results need to be confirmed on larger numbers of patients for each group and need to be studied with other potential co-occurring genes affected, such as* FLT3-ITD*. 

In conclusions, we report here for the first time two novel *NPM1* mutations that were identified in adult Syrian CN-AML patients. Although the prognostic value of these non-A types of *NPM1* mutations requires further investigation, the incidence of *NPM1* mutations in adult Syrian AML patients was similar to that reported by other studies. High WBC, dominant M4 and/or M5 subtypes, a better OS and CR characterized *NPM1mut *patients in our study. Molecular assessment of *NPM1* mutation at diagnosis offers valuable additional prognostic information and may there by markedly affect therapeutic decisions. 
